# A Multi-Task Training Semantic Communication System for Image Reconstruction and Classification Tasks

**DOI:** 10.3390/jimaging12080367

**Published:** 2026-08-08

**Authors:** Zijun Wang, Hongcheng Li

**Affiliations:** Air Traffic Control and Navigation College, Air Force Engineering University, Xi’an 710051, China; lihongcheng22@nudt.edu.cn

**Keywords:** semantic communication, wireless image transmission, image reconstruction, image classification, multi-task learning, task-oriented communication

## Abstract

Semantic communication provides a task-oriented alternative to conventional bit-level transmission. For wireless image transmission, existing systems mainly optimize image reconstruction, while downstream classification is often handled by a separate model. This paper proposes Mission-ADWITT, a multi-task semantic communication framework that extends the ADWITT backbone with a classification branch and jointly optimizes image reconstruction and classification. To improve joint training, the reconstruction backbone is initialized from a pretrained ADWITT-CIFAR10 model, while the classification branch is randomly initialized and fine-tuned together with the backbone. Experiments are conducted on CIFAR-10 over AWGN channels at SNR values of 0, 5, 10, 15, and 20 dB. Compared with Mission-ADWITT without ADWITT initialization, the baseline-initialized model improves average classification accuracy from 75.156% to 82.988%, PSNR from 27.933 dB to 30.828 dB, and MS-SSIM from 0.9637 to 0.9804. It also achieves classification accuracy comparable to the separate ADWITT + ResNet18 baseline, although reconstruction quality remains lower. These results highlight the importance of reconstruction-pretrained initialization and the trade-off between visual fidelity and semantic performance.

## 1. Introduction

In recent years, deep learning-based joint source-channel coding (JSCC) has become an important research direction for wireless image transmission and semantic communication. Different from conventional communication systems that separately optimize source coding and channel coding, deep JSCC learns an end-to-end mapping from source data to channel symbols and directly optimizes the reconstruction objective under channel distortion [1,2,3,4,5]. For image sources, this paradigm has shown strong potential because visual information is highly structured and can be compressed into compact latent representations. Existing image semantic communication systems have further extended this idea by introducing nonlinear transform coding, task-oriented semantic features, and adaptive transmission strategies to improve robustness under different channel conditions [6,7,8,9,10,11,12].

Most existing image semantic communication studies, however, still focus primarily on image reconstruction quality. In these systems, the receiver reconstructs the image first, and downstream vision tasks, such as classification or recognition, are usually performed by an additional model after reconstruction. This separated design is straightforward, but it does not explicitly optimize the interaction between pixel-level reconstruction fidelity and task-level semantic utility. In practical intelligent communication scenarios, the receiver may need both a visually meaningful reconstructed image and a reliable semantic decision. Therefore, evaluating a semantic communication system only from the reconstruction perspective may be insufficient.

Recent studies in imaging and visual data processing also indicate that reconstruction and downstream visual analysis are increasingly coupled. For example, deep learning and clustering models have been used to improve the quality of 3D point-cloud reconstruction under noisy acquisition conditions [13]. Computational imaging studies, such as non-line-of-sight imaging, further show that reconstruction performance is closely related to the imaging setting and physical constraints of data acquisition [14]. In addition, multispectral image analysis for wildfire detection demonstrates the importance of task-oriented visual interpretation in modern imaging systems [15]. Although these studies address different imaging scenarios, they collectively suggest that visual reconstruction should not be considered in isolation from downstream semantic tasks. This observation motivates the development of semantic communication models that jointly consider image recovery and task-level recognition.

To address this issue, this paper proposes Mission-ADWITT, a multi-task semantic communication framework for joint image reconstruction and classification. The proposed model is built on the ADWITT wireless image transmission backbone and extends the receiver with a ResNet18-based classification branch [16]. The goal is not to replace the separately optimized ADWITT reconstruction model, but to investigate whether a unified semantic communication model can support both reconstruction and classification within the same receiver-side framework. In this design, the reconstruction branch preserves image fidelity, while the classification branch encourages the transmitted representation to retain task-relevant semantic information.

A key challenge in this setting is that directly training a joint reconstruction–classification model may lead to unstable optimization and limited generalization, especially when the reconstruction backbone is not sufficiently initialized. Therefore, in the revised Mission-ADWITT training strategy, we adopt reconstruction-pretrained initialization. Specifically, the encoder–decoder reconstruction backbone is initialized from a pretrained ADWITT-CIFAR10 model, while the classification branch is randomly initialized and jointly fine-tuned under the multi-task objective. This strategy changes only the parameter initialization and does not modify the network architecture, channel model, or loss formulation. The ablation results show that this initialization is important for stable multi-task optimization.

The main contributions of this paper are summarized as follows:

First, we formulate wireless image semantic communication as a joint reconstruction–classification problem. Instead of treating image reconstruction and classification as two isolated stages, the proposed framework explicitly considers both pixel-level fidelity and task-level semantic utility.

Second, we develop Mission-ADWITT, a unified receiver-side framework that combines an ADWITT-based image reconstruction backbone with a ResNet18-based classification branch. This framework enables the receiver to perform image reconstruction and classification simultaneously.

Third, we introduce a reconstruction-pretrained initialization strategy for Mission-ADWITT. The pretrained ADWITT reconstruction backbone is loaded into the corresponding reconstruction modules of Mission-ADWITT, while the classification branch is trained jointly from random initialization. This strategy substantially improves the performance of the joint model compared with Mission-ADWITT without ADWITT initialization.

Fourth, we provide full CIFAR-10 test-set evaluations under AWGN channels at SNR values of 0, 5, 10, 15, and 20 dB. The revised experiments report exact numerical results in terms of classification accuracy, PSNR, MS-SSIM, loss, and delay. We also include a separate ADWITT + ResNet18 baseline and an ablation study comparing Mission-ADWITT with and without ADWITT initialization.

The rest of this paper is organized as follows. Section 2 introduces the semantic communication system architecture, the ADWITT backbone, and the evaluation metrics. Section 3 describes the Mission-ADWITT architecture, the multi-task loss formulation, and the reconstruction-pretrained initialization strategy. Section 4 presents the experimental settings, quantitative results, and ablation analysis. Section 5 discusses the limitations and the reconstruction–classification trade-off. Section 6 concludes the paper.

## 2. Background and Evaluation Metrics

### 2.1. Semantic Communication System Architecture

**Figure 1:**
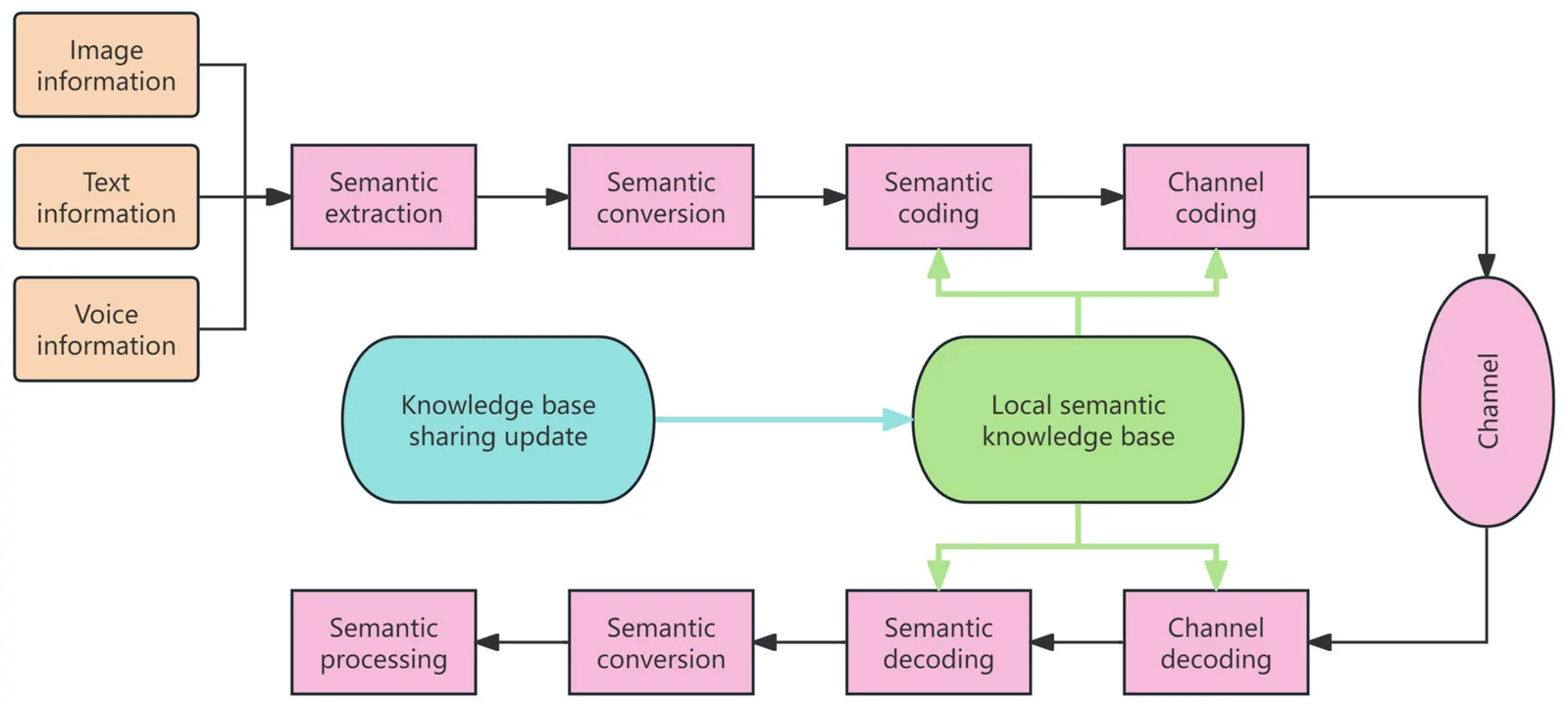
The architecture of the semantic communication system model.

Figure 1 shows the general architecture of a semantic communication system. Different from conventional communication systems that mainly pursue bit-level recovery, semantic communication emphasizes the extraction, transmission, and recovery of task-relevant information. At the transmitter, the source data are first analyzed according to the target task, and compact semantic features are extracted and encoded for channel transmission. After passing through the wireless channel, the receiver decodes the transmitted features and reconstructs the information required by downstream applications.

In this framework, the transmitter and receiver are usually assumed to share a consistent semantic knowledge base or task-oriented representation space, which helps maintain semantic consistency during transmission [17,18,19,20,21]. For image sources, this task-oriented perspective is particularly important because downstream applications often require not only visual reconstruction but also semantic understanding, such as object recognition or image classification. Therefore, semantic communication provides a suitable foundation for jointly considering image reconstruction and classification within a unified transmission system.

### 2.2. ADWITT Backbone for Wireless Image Transmission

ADWITT is an adaptive wireless image transmission transformer architecture designed for image transmission and reconstruction under wireless channel conditions [22]. It adopts a Swin-Transformer-based encoder–decoder structure and introduces a channel adaptive module to improve robustness under different signal-to-noise ratios (SNRs). The channel adaptive module is related to the variational approximation and information bottleneck principle [23], and it adjusts transmitted image features through modulation mechanisms according to channel state information [24].

**Figure 2:**
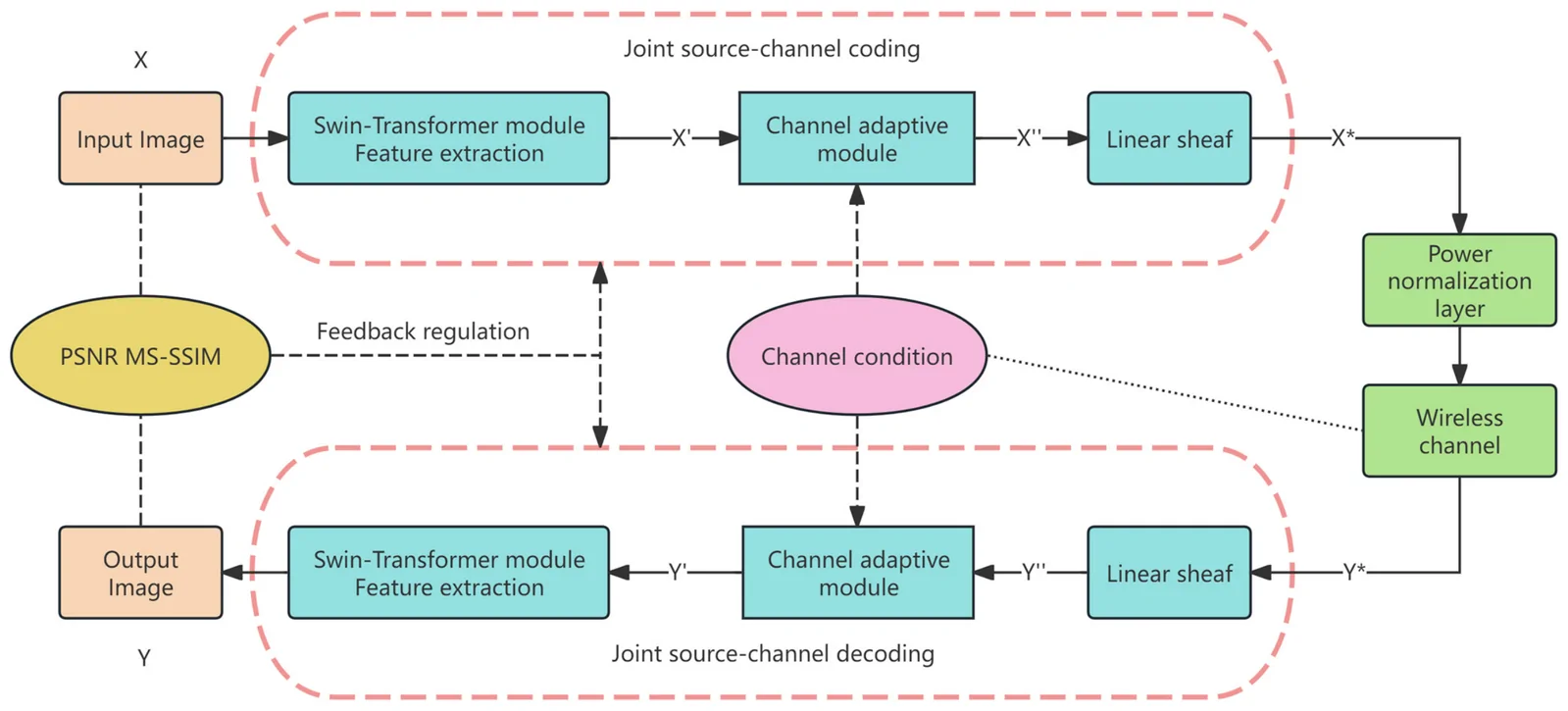
Architecture of the ADWITT model.

As illustrated in Figure 2, the input image is first transformed into latent feature representations by the encoder. These features are then processed by the channel-adaptive module and constrained by the power normalization layer before being transmitted through the wireless channel. At the receiver, the decoder reconstructs the image from the received features. Since ADWITT is trained in an end-to-end manner, the encoder, channel adaptation module, channel transmission process, and decoder can be jointly optimized for image reconstruction. Owing to its reconstruction capability and adaptability to varying channel conditions, ADWITT is selected as the reconstruction backbone of Mission-ADWITT in this study.

### 2.3. Evaluation Metrics

This study evaluates the proposed system from both image reconstruction and image classification perspectives. PSNR and MS-SSIM are used to evaluate image reconstruction quality, while classification accuracy is used to evaluate task-level semantic performance. The composite multi-task loss and average inference delay are additionally reported as auxiliary indicators of optimization behavior and computational efficiency.

For image reconstruction, Peak Signal-to-Noise Ratio (PSNR) is used to quantify the pixel-level fidelity of the reconstructed images. PSNR is derived from the mean square error (MSE) between the original image and the reconstructed image and is widely used in image reconstruction and image super-resolution studies [25,26]:

(1) $$PSNR=10\times \log_{10}\left(\frac{{MAX}^{2}}{MSE}\right),$$

(2) $$MSE=\frac{1}{M\times N}\sum_{i=1}^{M}\sum_{j=1}^{N}{\left[I\left(i,j\right)-K\left(i,j\right)\right]}^{2}$$

where MAX denotes the maximum possible pixel value of the image, and MSE represents the mean square error between the original image and the reconstructed image. A higher PSNR value indicates lower average pixel-wise distortion and better reconstruction fidelity. However, because PSNR is based on pixel-level errors, it does not directly model all aspects of human visual perception and should therefore be interpreted together with a structural similarity metric.

In addition to PSNR, this study reports the Multi-Scale Structural Similarity Index Measure (MS-SSIM), which extends structural similarity assessment across multiple image scales [27]. MS-SSIM evaluates luminance, contrast, and structural consistency between the original and reconstructed images and therefore provides information that is complementary to pixel-wise distortion metrics. The combined use of PSNR and structural-similarity-based metrics is common in image reconstruction evaluation [25,26,27]. A higher MS-SSIM value indicates greater perceptual and structural similarity between the original and reconstructed images.

For the image classification task, overall classification accuracy is adopted as the main semantic performance metric. Accuracy is defined as the proportion of correctly predicted samples among all evaluated samples and is commonly reported as a summary measure in multiclass classification studies [28]:

(3) Accuracy=(N_correct/N_total)×100%,

where N_correct denotes the number of correctly classified samples, and N_total denotes the total number of test samples. In the context of semantic image transmission, classification accuracy reflects whether the information preserved through the wireless transmission and reconstruction process remains sufficient to support reliable downstream semantic decision-making.

The overall multi-task loss reported in this study is not treated as a universally standardized external performance metric. Instead, it is the study-specific composite optimization objective defined in Section 3.4. Weighted combinations of task-specific objectives are commonly used as baseline formulations in multi-task learning, although the relative loss weights can substantially affect the optimization process [29,30,31,32]. Therefore, the reported loss values are used only to compare Mission-ADWITT variants that share the same composite objective. Since the separate ADWITT + ResNet18 baseline does not use the same joint loss formulation, its loss value is not directly compared with those of the Mission-ADWITT variants.

The average inference delay is reported as an auxiliary indicator of computational efficiency. It is interpreted together with the model structure and task setting rather than as the sole measure of deployment complexity.

## 3. The Mission-ADWITT Model

The Mission-ADWITT model is designed to support two receiver-side objectives in wireless image semantic communication: image reconstruction and image classification. Unlike a separated pipeline in which an image is first reconstructed by a semantic communication model and then classified by an independent classifier, Mission-ADWITT introduces a unified multi-task framework in which the reconstruction branch and the classification branch are optimized jointly. The purpose of this design is to preserve both pixel-level visual information and task-level semantic information during transmission.

The proposed framework is built upon the ADWITT wireless image transmission backbone. ADWITT provides the encoder, channel adaptive module, power normalization operation, wireless channel interface, and decoder for image reconstruction. A ResNet18-based classification branch is further incorporated at the receiver side to predict the image category from the received/reconstructed representation. Therefore, the receiver can generate both a reconstructed image and a classification result within the same semantic communication system.

### 3.1. Multi-Task Training Strategy

Multi-task learning aims to improve model efficiency and generalization by training related tasks within a shared framework [29,30]. In the context of image semantic communication, reconstruction and classification are related but not identical objectives. Image reconstruction emphasizes pixel-level fidelity, while image classification emphasizes task-relevant semantic discrimination. If these two tasks are optimized completely separately, the communication system may preserve visual details that are less useful for recognition, or it may fail to explicitly encourage the transmitted representation to retain discriminative semantic information.

**Figure 3:**
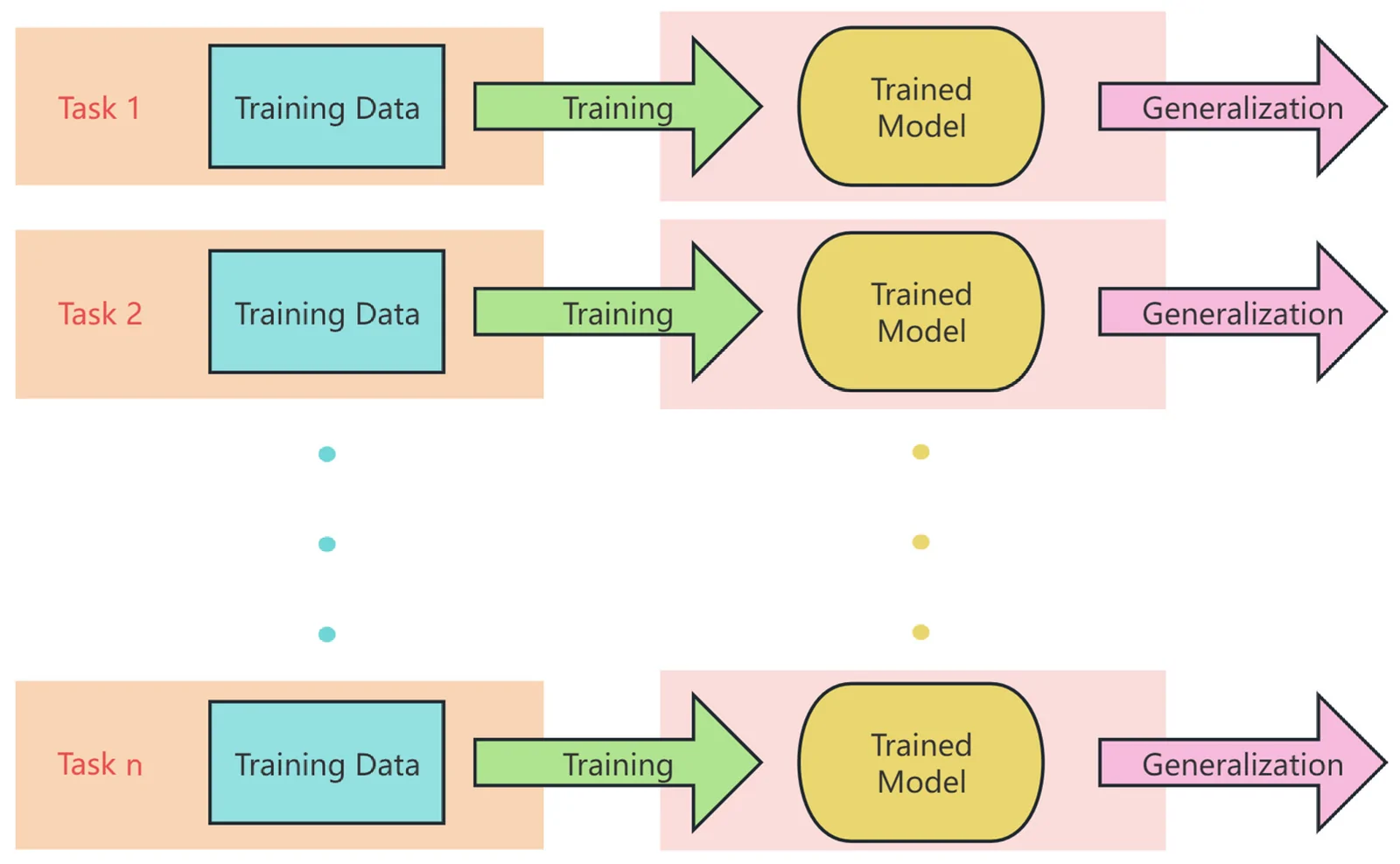
Single-task learning flowchart.

**Figure 4:**
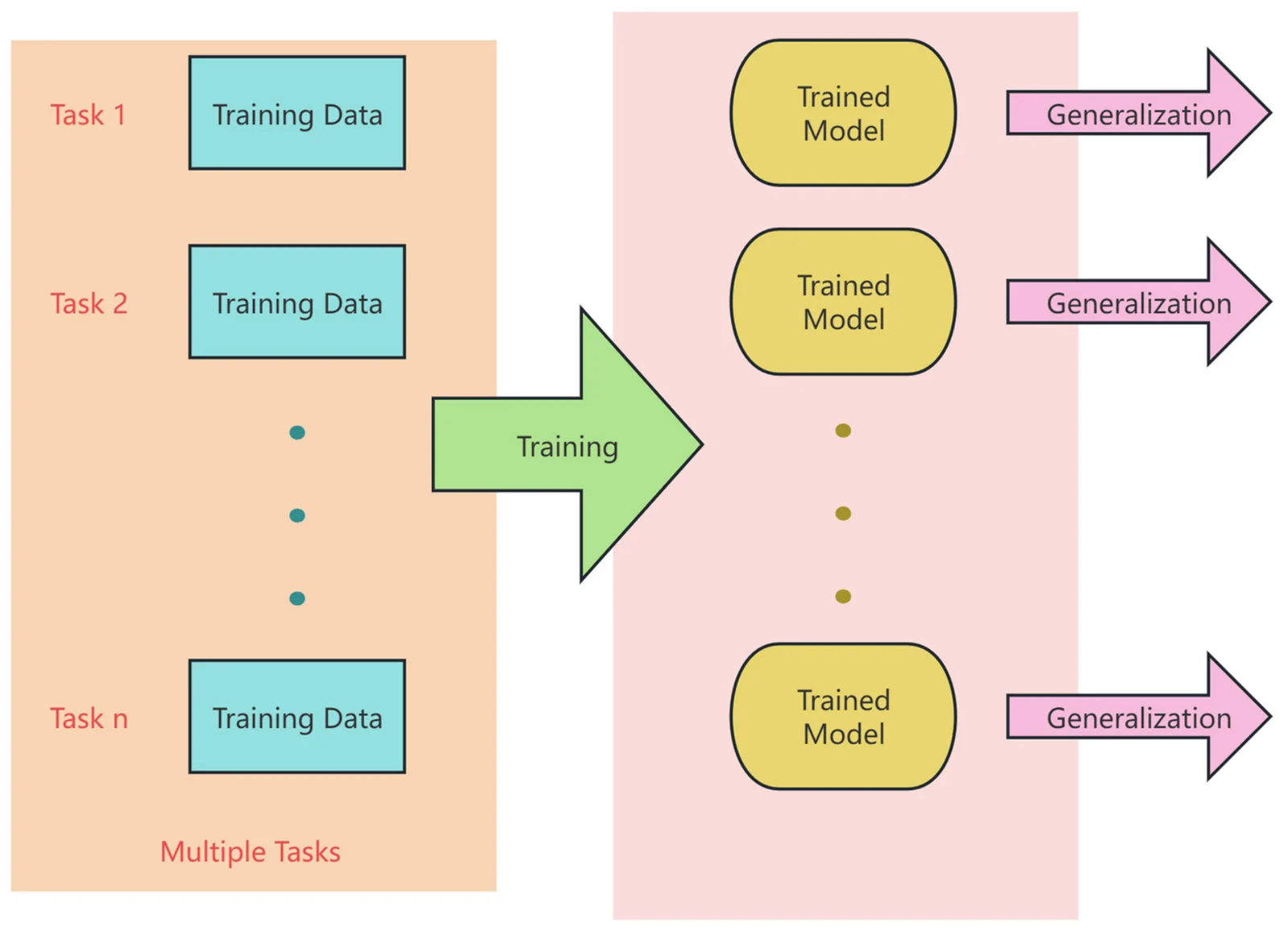
Multi-task learning flowchart.

Figure 3 and Figure 4 illustrate the difference between single-task learning and multi-task learning. In single-task learning, each task is trained independently with its own model and objective. In contrast, multi-task learning allows related tasks to share part of the representation while retaining task-specific optimization objectives.

**Figure 5:**
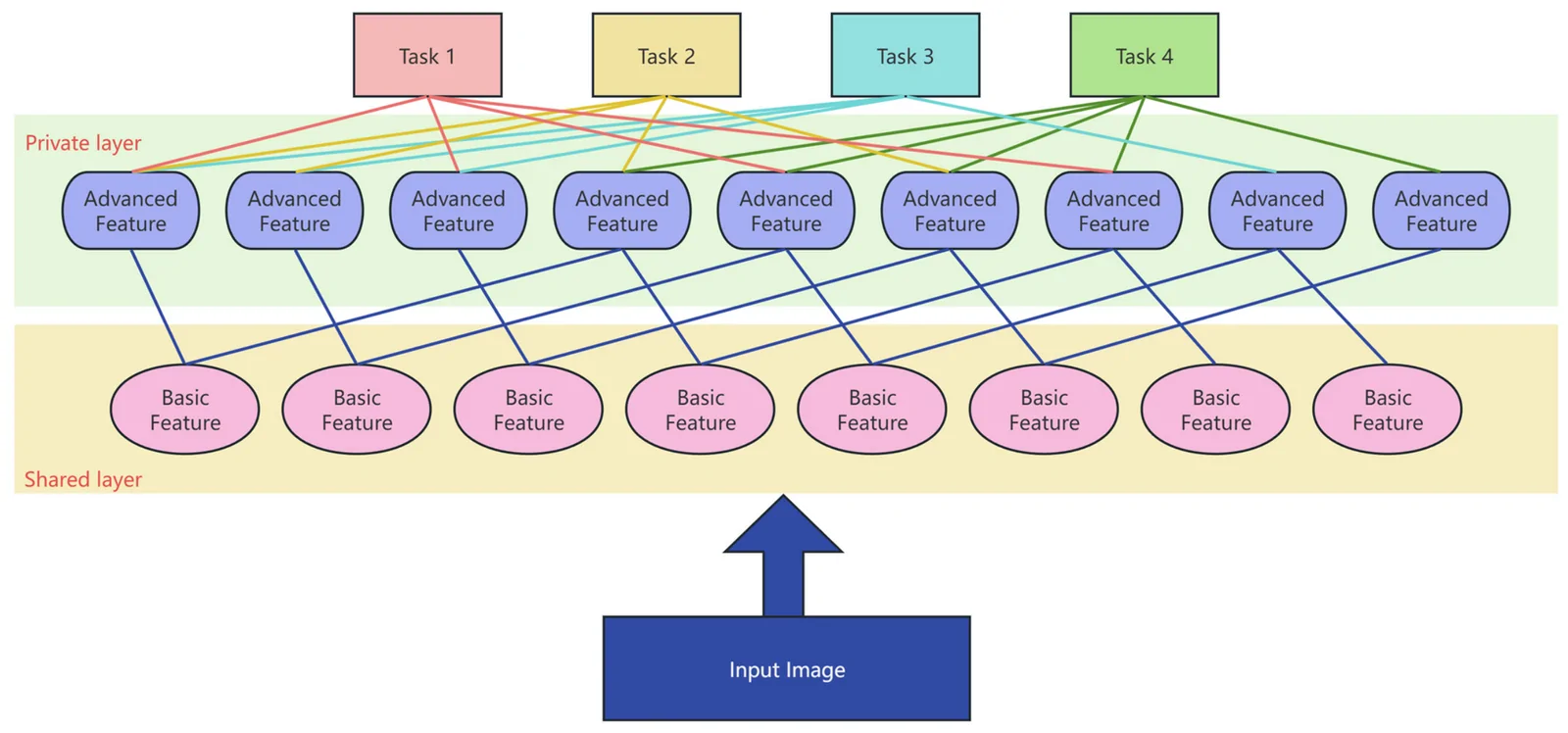
Overview of multi-task learning neural network architecture.

As shown in Figure 5, a typical multi-task network contains shared layers and task-specific branches. The shared layers extract common representations from the input, while each task branch optimizes its own objective [31]. This structure has been widely used in multi-task learning because it can reduce redundant computation and allow useful information to be transferred across tasks. At the same time, the relative weights of different task losses must be carefully controlled; otherwise, one task may dominate the optimization process and degrade the other task [32].

In Mission-ADWITT, the shared part is mainly the ADWITT-based semantic transmission and reconstruction backbone. The reconstruction objective encourages the transmitted features to preserve visual fidelity, while the classification objective encourages the receiver-side representation to retain semantic information useful for recognition. This formulation makes the model suitable for intelligent image transmission scenarios where both reconstructed image quality and downstream classification performance are required.

### 3.2. Mission-ADWITT System Design

**Figure 6:**
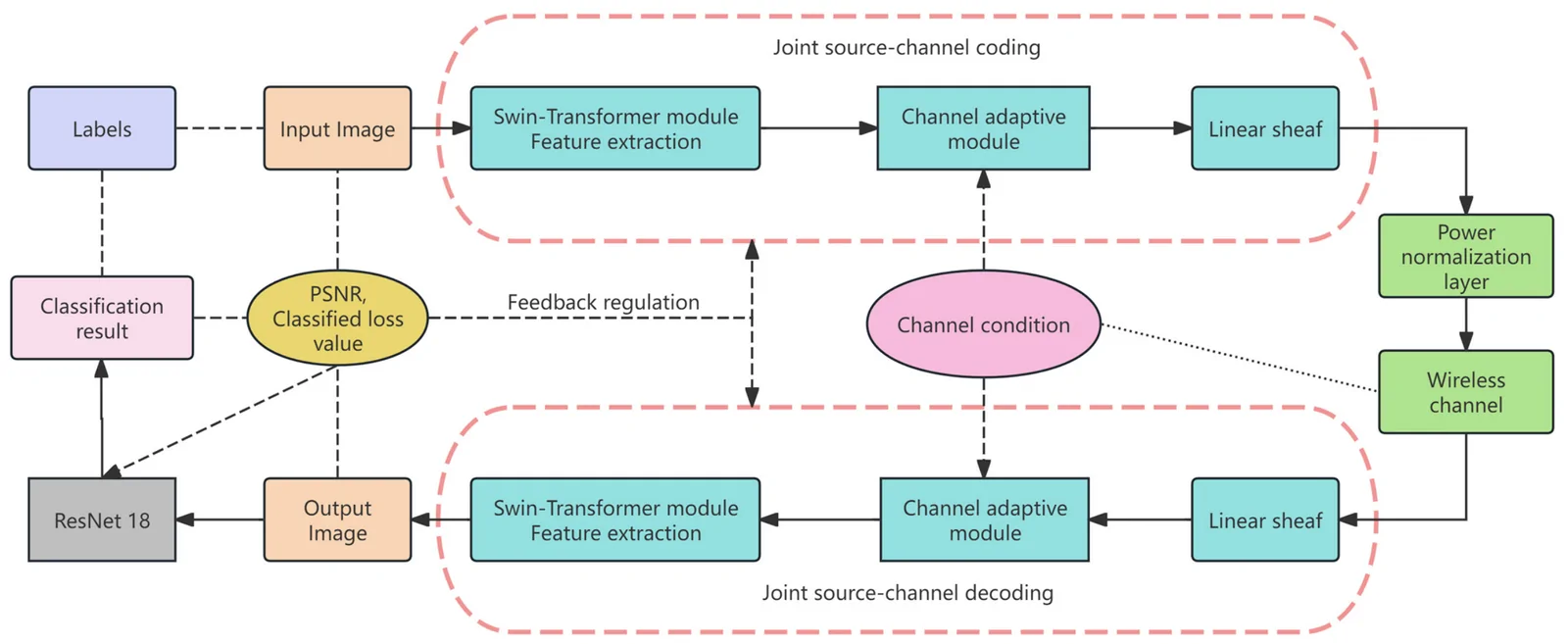
Architecture of the Mission-ADWITT model.

Figure 6 shows the overall architecture of Mission-ADWITT. At the transmitter, the input image is encoded by the ADWITT encoder and then processed by the channel-adaptive module. The encoded representation is normalized and transmitted through the wireless channel. At the receiver, the decoder reconstructs the image from the received representation. In addition to the reconstruction output, a ResNet18-based classification branch is introduced to predict the category label.

The architecture can be interpreted as a unified receiver-side reconstruction–classification framework. The reconstruction branch produces the recovered image and is evaluated using PSNR and MS-SSIM, while the classification branch produces the predicted class label and is evaluated using classification accuracy. Since the two objectives are optimized in the same training framework, the model can jointly consider visual fidelity and semantic discriminability.

It should be noted that the proposed model is an incremental extension of the ADWITT image transmission framework rather than a completely new communication architecture. The main contribution lies in formulating image reconstruction and classification as coupled objectives in a semantic communication system, integrating a task-oriented classification branch into the ADWITT receiver, and examining how initialization and multi-task optimization affect the reconstruction–classification trade-off.

### 3.3. Reconstruction-Pretrained Initialization

Directly training the joint Mission-ADWITT model without a well-initialized reconstruction backbone may lead to limited generalization. In the initial training setting, the Mission-ADWITT checkpoint trained without ADWITT reconstruction initialization showed noticeably weaker test performance than the separately optimized ADWITT + ResNet18 baseline. This suggests that the reconstruction backbone plays a critical role in stabilizing the joint optimization of reconstruction and classification.

To address this issue, the revised training strategy adopts reconstruction-pretrained initialization. Specifically, the encoder–decoder reconstruction backbone of Mission-ADWITT is initialized from a pretrained ADWITT-CIFAR10 model trained for wireless image reconstruction under the AWGN channel. The corresponding reconstruction parameters are loaded into the ADWITT reconstruction modules of Mission-ADWITT. The classification branch is not included in the ADWITT reconstruction checkpoint and is therefore randomly initialized.

After initialization, the entire Mission-ADWITT model is jointly fine-tuned using the same multi-task objective. This strategy changes only the parameter initialization. It does not modify the network architecture, channel model, reconstruction branch, classification branch, or loss formulation. The purpose is to provide the joint model with a stable reconstruction-oriented starting point before classification-oriented semantic optimization is introduced.

This initialization strategy is also used as an ablation factor in the experiments. The Mission-ADWITT model without ADWITT initialization is treated as the without-initialization variant, while the baseline-initialized Mission-ADWITT is treated as the final proposed variant in the revised experiments.

### 3.4. Multi-Task Loss Function

The Mission-ADWITT model is optimized using a composite loss function that combines a reconstruction-related term and a classification-related term. Weighted combinations of task-specific objectives constitute a commonly used baseline strategy in multi-task learning, while previous studies have also shown that the relative weighting of the task losses can materially affect joint optimization [29,30,31,32]. The classification term, denoted as Loss2, is measured by cross-entropy loss between the predicted class distribution and the ground-truth label. A lower cross-entropy loss indicates better classification prediction.

For the reconstruction task, PSNR is used as the primary reconstruction metric. Since PSNR is measured in decibels and cannot be directly minimized on the same numerical scale as cross-entropy, the PSNR-related term is transformed into a normalized reconstruction loss. In this study, the reconstruction loss is written as follows:

(4) $$Loss1=0.5-\left(PSNR-30\right)/100$$

The value of 30 dB is used as a fixed reference point to center the PSNR-related term rather than as a universal perceptual threshold. The constant 0.5 provides an offset for the transformed reconstruction term, while division by 100 reduces the numerical scale of PSNR relative to the cross-entropy loss. The outer factor of 100 in Equation (5) rescales the combined objective for optimization and reporting without changing the relative task weight determined by a. These settings are empirical engineering choices rather than theoretically optimal values.

The total loss is formulated as follows:

(5) $$Loss=100\times \left(a\times Loss1+\left(1-a\right)\times Loss2\right)$$

where the weighting coefficient controls the trade-off between reconstruction fidelity and classification accuracy. In the revised CIFAR-10 experiments, the coefficient is set to 0.5 so that the reconstruction and classification terms are given balanced importance during joint fine-tuning. The choice of this coefficient is empirical, and the results should be interpreted as the performance of a balanced reconstruction–classification setting rather than as proof that this coefficient is globally optimal. Accordingly, the term “multi-task loss” in this paper refers to the study-specific optimization objective defined in Equation (5), rather than to a universally standardized evaluation metric. The PSNR transformation, reference value, scaling constants, and fixed weighting coefficient are empirical design choices of the present implementation. Consequently, the numerical loss values are only interpreted through controlled comparisons between Mission-ADWITT variants trained using the same formulation.

This formulation allows Mission-ADWITT to jointly optimize image fidelity and classification performance. However, it also introduces an inherent trade-off: increasing the emphasis on reconstruction may improve PSNR or MS-SSIM but may not necessarily improve classification accuracy, while increasing the emphasis on classification may preserve more task-discriminative features at the cost of reconstruction quality. This trade-off is further discussed in the experimental analysis and conclusion.

## 4. Experiments and Results

### 4.1. Experimental Settings

The revised experiments focus on CIFAR-10 to provide a complete and controlled comparison among the separate baseline, the Mission-ADWITT variant without ADWITT initialization, and the baseline-initialized Mission-ADWITT model. CIFAR-10 contains 60,000 color images from 10 classes, including 50,000 training images and 10,000 test images [33]. In the final evaluation, the full CIFAR-10 test set is used at each SNR value.

All experiments are conducted under the AWGN channel. The evaluated SNR values are 0, 5, 10, 15, and 20 dB. The channel bandwidth parameter is set to C = 192. The multi-task loss coefficient is set to a = 0.5, corresponding to a balanced reconstruction–classification setting. The baseline-initialized Mission-ADWITT model is fine-tuned for 3 epochs with a batch size of 128 using the Adam optimizer and a learning rate of 0.0001. The best checkpoint is selected according to the highest accuracy during epoch-wise evaluation; ties are resolved by higher PSNR and then lower loss. The experimental settings are summarized in Table 1.

### 4.2. Compared Methods

To clarify the contribution of the proposed training strategy, three methods are compared in the revised experiments.

First, the separate ADWITT + ResNet18 baseline uses a pretrained ADWITT reconstruction model for wireless image reconstruction and a ResNet18 classifier for classification. This baseline represents a separated reconstruction-then-classification pipeline. Since this baseline does not share the same joint multi-task loss as Mission-ADWITT, its loss value is not directly compared with those of the Mission-ADWITT variants.

Second, Mission-ADWITT without ADWITT initialization denotes the joint reconstruction–classification model trained without loading the pretrained ADWITT reconstruction backbone. This variant is used as an ablation baseline to evaluate whether direct joint training is sufficient for stable multi-task optimization.

Third, Mission-ADWITT with ADWITT initialization is the proposed revised model. Its reconstruction backbone is initialized from the pretrained ADWITT-CIFAR10 model, while the classification branch is randomly initialized and jointly fine-tuned. This variant is used as the main Mission-ADWITT result in the revised manuscript.

The roles of the compared methods are summarized in Table 2.

### 4.3. Overall Quantitative Comparison

Table 3 reports the average performance over SNR = 0, 5, 10, 15, and 20 dB. Compared with the Mission-ADWITT variant without ADWITT initialization, the baseline-initialized Mission-ADWITT achieves a clear improvement across all major metrics. The average classification accuracy increases from 75.156% to 82.988%, the average PSNR increases from 27.933 dB to 30.828 dB, and the average MS-SSIM increases from 0.9637 to 0.9804. The average loss is also reduced from 102.970 to 58.883.

Compared with the separate ADWITT + ResNet18 baseline, the baseline-initialized Mission-ADWITT achieves comparable classification accuracy. Specifically, the average classification accuracy is 82.988% for the proposed model and 82.940% for the separate baseline. However, the reconstruction quality of the proposed model remains lower than that of the separately optimized ADWITT reconstruction baseline. The average PSNR of the separate baseline is 42.209 dB, while that of the baseline-initialized Mission-ADWITT is 30.828 dB. Similarly, the average MS-SSIM of the separate baseline is 0.9969, whereas that of the proposed model is 0.9804. These results indicate that the unified multi-task model can achieve classification performance comparable to the separate baseline, but there is still a reconstruction-quality gap compared with the reconstruction-specialized ADWITT model.

The per-SNR results are further reported in Table 4. For the baseline-initialized Mission-ADWITT, the classification accuracy remains stable across different SNR values, increasing from 82.44% at 0 dB to 83.21% at 20 dB. The reconstruction metrics also improve as the channel condition becomes better, with PSNR increasing from 28.932 dB at 0 dB to 31.643 dB at 20 dB and MS-SSIM increasing from 0.9678 to 0.9853. This trend is consistent with the expected behavior of wireless image transmission systems under increasing SNR.

### 4.4. Accuracy, Reconstruction Quality, Loss, and Delay Curves

**Figure 7:**
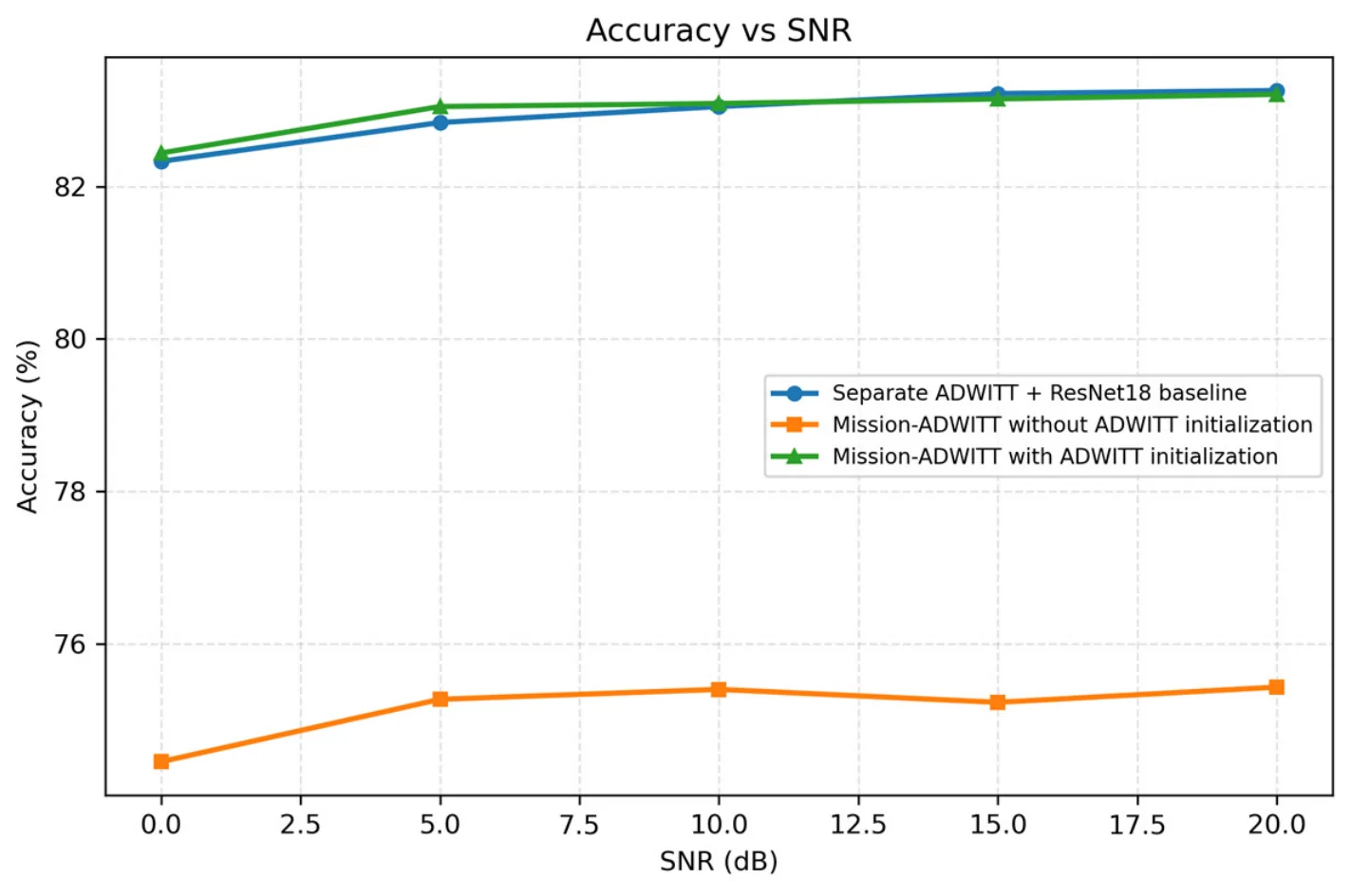
Classification accuracy under different SNR values on CIFAR-10.

Figure 7 shows the classification accuracy under different SNR values. The baseline-initialized Mission-ADWITT substantially improves the accuracy compared with Mission-ADWITT without ADWITT initialization and reaches a level comparable to the separate ADWITT + ResNet18 baseline. This result suggests that ADWITT reconstruction backbone initialization helps the joint model retain semantic information useful for classification.

**Figure 8:**
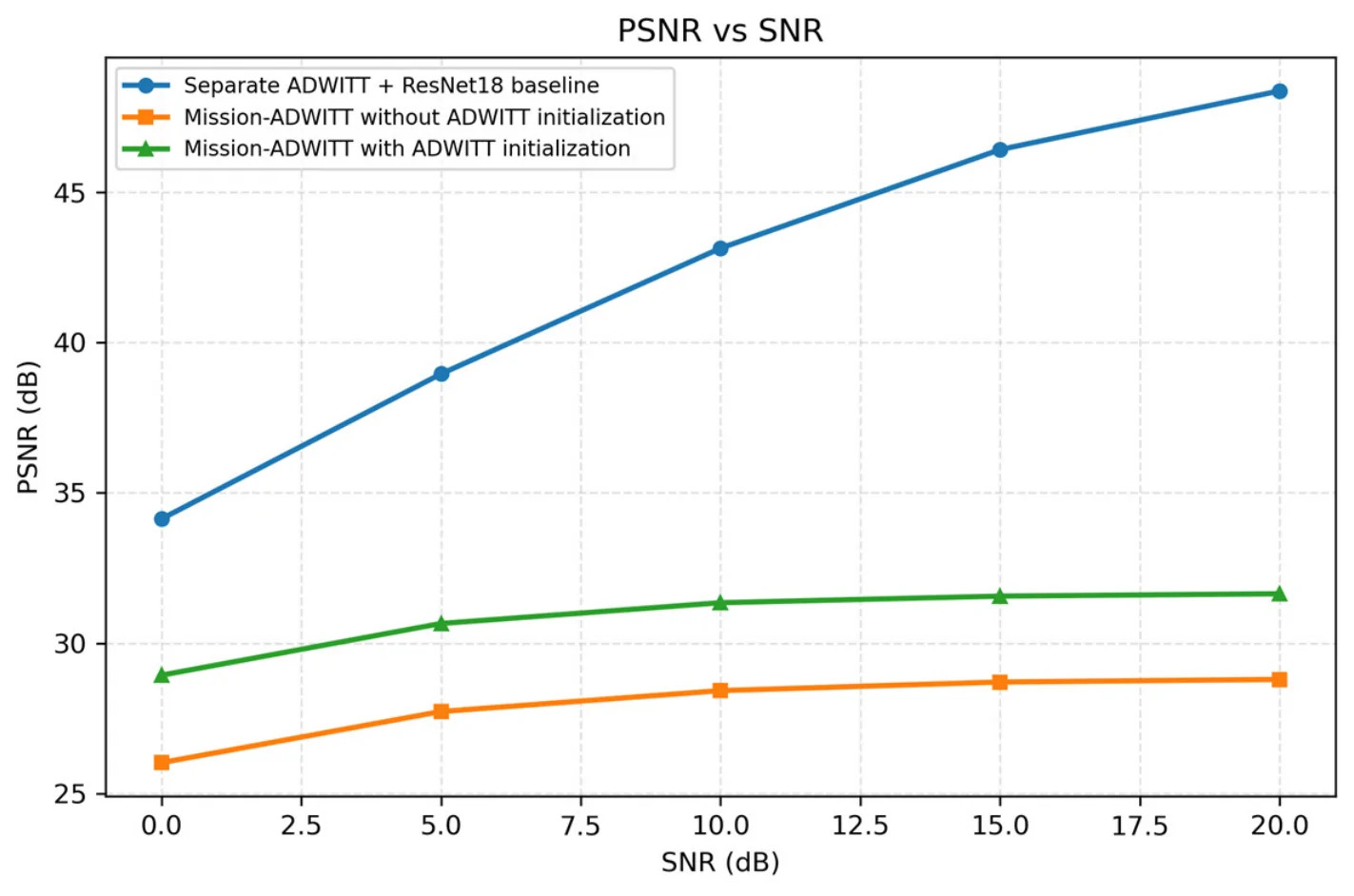
PSNR under different SNR values on CIFAR-10.

Figure 8 shows the PSNR comparison. The separate ADWITT baseline achieves the highest PSNR because it is optimized specifically for image reconstruction. The baseline-initialized Mission-ADWITT improves PSNR compared with the variant without initialization, but it does not reach the reconstruction quality of the separate ADWITT baseline. This result indicates that joint reconstruction–classification optimization introduces a reconstruction–classification trade-off.

**Figure 9:**
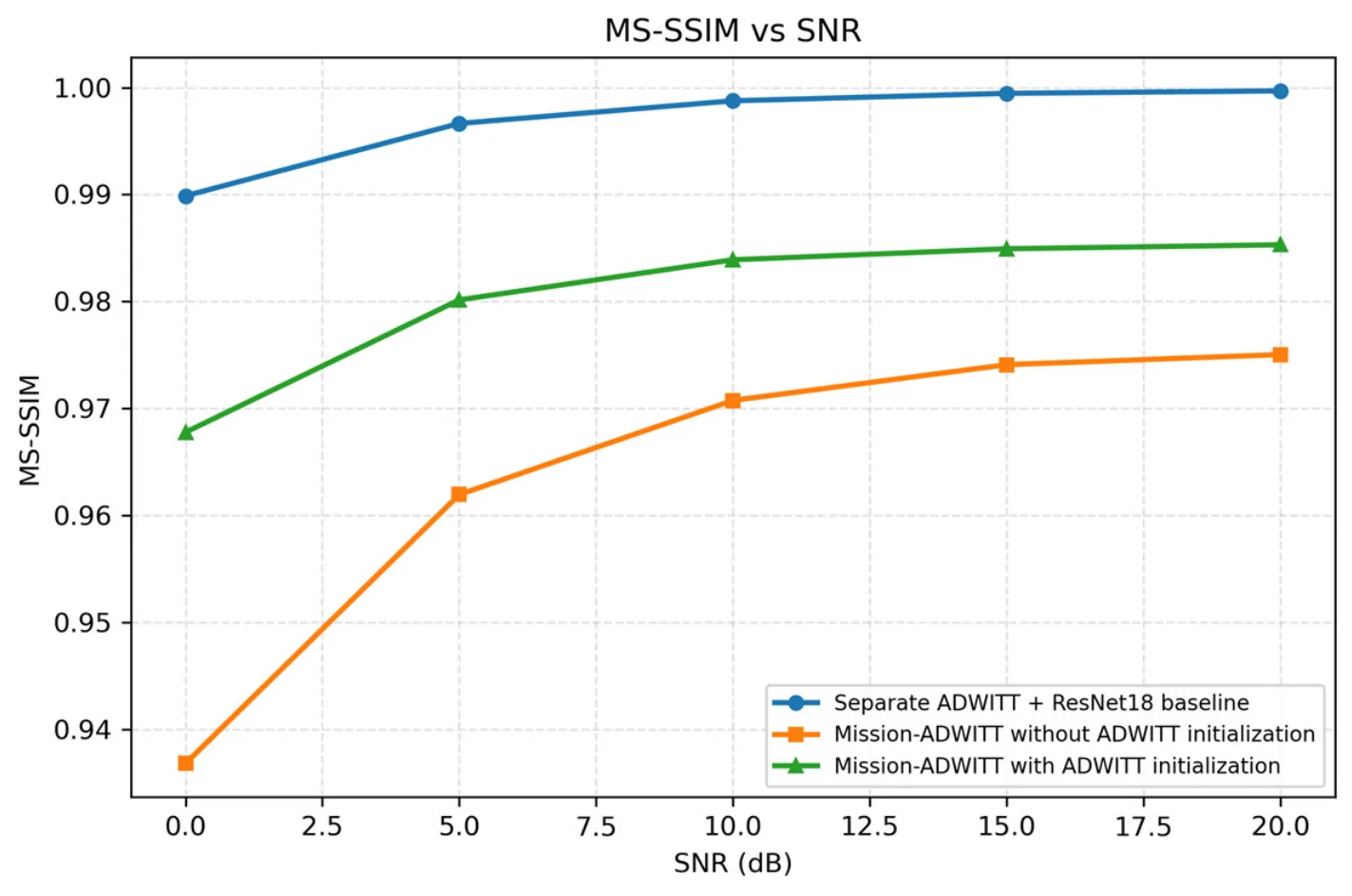
MS-SSIM under different SNR values on CIFAR-10.

Figure 9 reports MS-SSIM under different SNR values. The trend is consistent with the PSNR comparison. The baseline-initialized Mission-ADWITT improves structural similarity compared with the without-initialization variant, while the separate ADWITT baseline still achieves the best reconstruction quality. Therefore, the MS-SSIM results further confirm that the proposed initialization strategy improves the multi-task model but does not eliminate the reconstruction gap relative to the reconstruction-specialized baseline.

**Figure 10:**
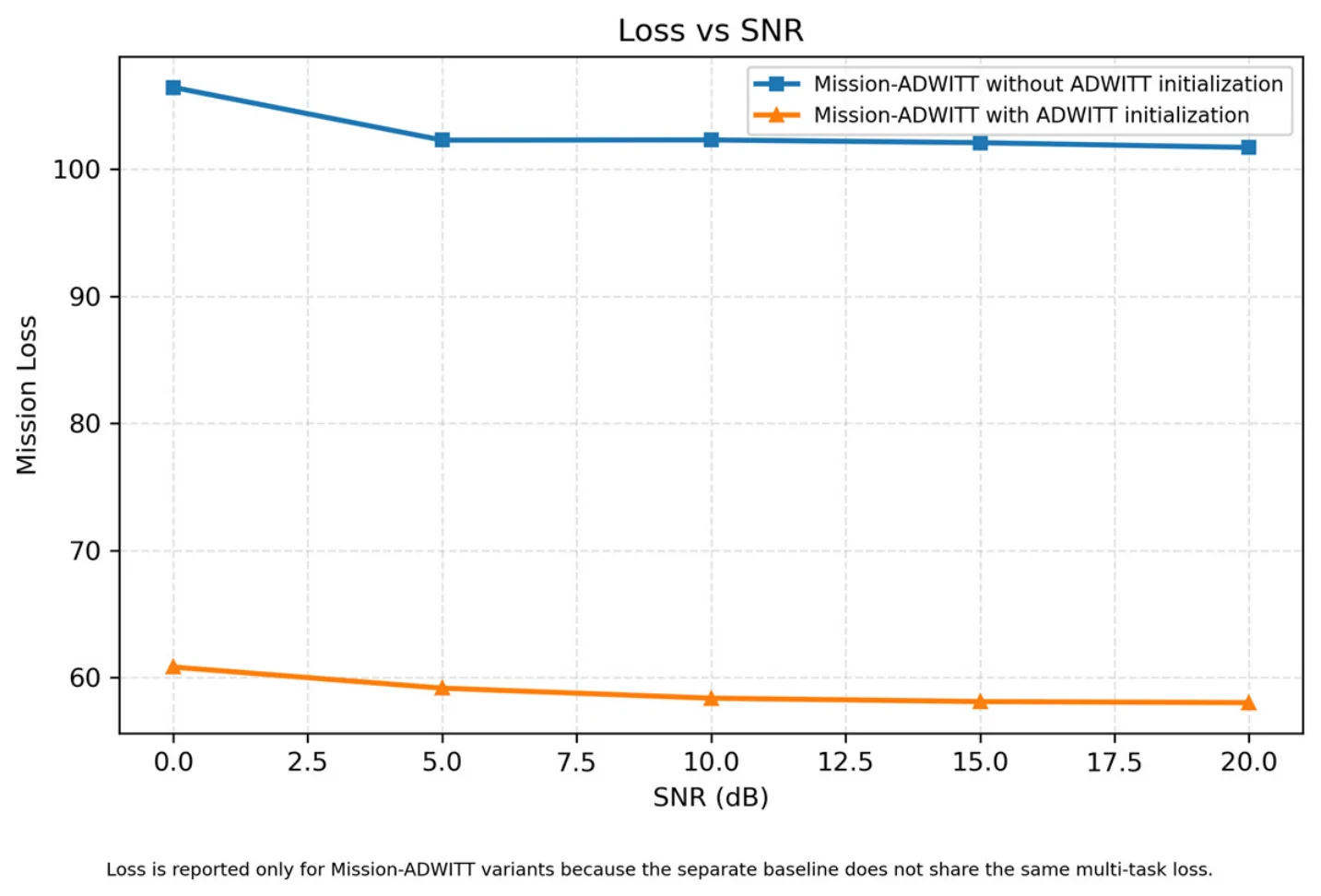
Multi-task loss under different SNR values on CIFAR-10.

Figure 10 presents the loss values of the two Mission-ADWITT variants. The separate ADWITT + ResNet18 baseline is not included in this figure because it does not use the same joint multi-task loss. The baseline-initialized Mission-ADWITT consistently obtains lower loss than the without-initialization variant across all SNR values, indicating that reconstruction-pretrained initialization provides a more stable starting point for joint optimization.

**Figure 11:**
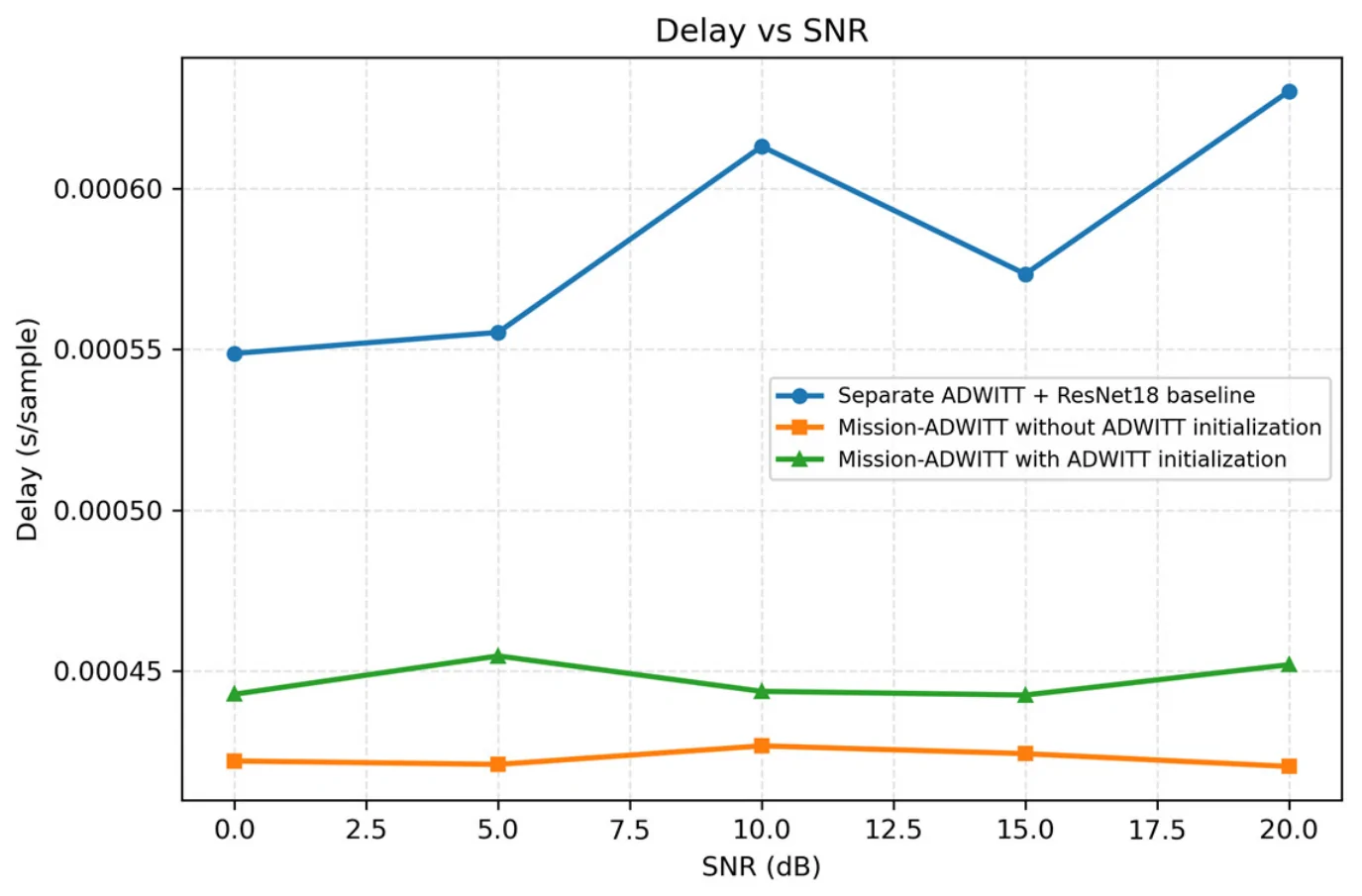
Average inference delay under different SNR values on CIFAR-10.

Figure 11 shows the average inference delay. The delay values of the compared methods remain within the same order of magnitude. Therefore, the delay results should be interpreted as an auxiliary efficiency indicator rather than as the sole evidence of deployment superiority. The main advantage of the baseline-initialized Mission-ADWITT lies in its unified reconstruction–classification framework and improved multi-task performance compared with the without-initialization variant.

### 4.5. Ablation Study on ADWITT Initialization

To evaluate the effect of ADWITT reconstruction backbone initialization, Table 5 compares Mission-ADWITT without ADWITT initialization and Mission-ADWITT with ADWITT initialization. The with-initialization variant improves the average classification accuracy by 7.832 percentage points, increases the average PSNR by 2.895 dB, and increases the average MS-SSIM by 0.0167. The average loss is reduced by 44.087.

These results show that the pretrained ADWITT reconstruction backbone is important for stable joint reconstruction–classification optimization. Without this initialization, the Mission-ADWITT model has limited test generalization, even though it is trained under the same architecture and loss formulation. This finding suggests that the improvement of the revised Mission-ADWITT model is not only due to adding a classification branch but also depends on providing a shared reconstruction backbone with strong initialization.

### 4.6. Summary of Experimental Findings

The revised experiments lead to three main observations. First, ADWITT reconstruction backbone initialization substantially improves Mission-ADWITT compared with the without-initialization variant. Second, the baseline-initialized Mission-ADWITT achieves classification accuracy comparable to the separate ADWITT + ResNet18 baseline within a unified reconstruction–classification framework. Third, the reconstruction quality of the proposed model remains lower than that of the separately optimized ADWITT reconstruction baseline, indicating that joint multi-task optimization still involves a trade-off between visual fidelity and semantic classification performance. This trade-off is further discussed in the next section.

## 5. Discussion

### 5.1. Effect of Reconstruction-Pretrained Initialization

The experimental results show that reconstruction-pretrained initialization is an important factor in the joint optimization of Mission-ADWITT. Compared with the Mission-ADWITT variant without ADWITT initialization, the baseline-initialized model achieves higher classification accuracy, PSNR, and MS-SSIM, together with a lower multi-task loss across all evaluated SNR values. Since the two Mission-ADWITT variants use the same network architecture, channel model, and loss formulation, the observed difference can mainly be attributed to the initialization of the reconstruction backbone.

A possible explanation is that the pretrained ADWITT backbone already provides a stable representation for wireless image reconstruction before the classification objective is introduced. Joint fine-tuning can therefore begin from a reconstruction-capable parameter space instead of simultaneously learning basic image recovery and semantic classification from an insufficiently initialized model. This reduces the optimization burden on the shared backbone and helps the classification branch learn task-relevant information without completely disrupting the reconstruction function.

These results do not imply that initialization alone constitutes a new semantic communication architecture. Rather, they indicate that parameter initialization should be treated as an important component of a multi-task semantic communication system design. In particular, directly combining reconstruction and classification branches does not necessarily guarantee effective joint optimization; the quality of the shared backbone before multi-task fine-tuning can substantially affect the final generalization performance.

### 5.2. Comparison with Representative Studies and the Separate Reconstruction–Classification Baseline

To further position the proposed framework within the existing literature, Table 6 provides a qualitative comparison with representative studies on deep joint source-channel coding, semantic-assisted image transmission, multi-task semantic communication, and adaptive wireless image reconstruction. A direct numerical comparison is not performed because the studies use different datasets, channel models, bandwidth settings, network architectures, and task objectives.

As summarized in Table 6, previous studies have separately investigated reconstruction-oriented deep JSCC, multi-level semantic assistance, multi-task image-feature transmission, and channel-adaptive image reconstruction. Mission-ADWITT differs from these studies by combining an ADWITT-based reconstruction backbone with a ResNet18 classification branch and by evaluating reconstruction fidelity and classification performance simultaneously within the same receiver-side framework. Nevertheless, the comparison in Table 6 is qualitative, and it should not be interpreted as evidence of comprehensive numerical superiority over the listed methods.

Within the controlled experiments conducted in this study, the baseline-initialized Mission-ADWITT achieves an average classification accuracy of 82.988%, which is comparable to the 82.940% obtained by the separate ADWITT + ResNet18 baseline. This result suggests that a unified reconstruction–classification framework can preserve classification-oriented semantic information at a level similar to that of a separately implemented pipeline.

However, the unified model does not outperform the separate baseline in reconstruction quality. The average PSNR and MS-SSIM of Mission-ADWITT remain lower than those of the separately optimized ADWITT reconstruction model. The separate ADWITT model is optimized specifically for image reconstruction, whereas Mission-ADWITT must simultaneously satisfy reconstruction and classification objectives through a shared training process. Consequently, part of the model capacity and optimization direction is allocated to classification-oriented semantic information rather than exclusively to pixel-level recovery.

Therefore, the main advantage of Mission-ADWITT should not be interpreted as comprehensive superiority over the separate baseline. Its contribution lies in demonstrating the feasibility of integrating reconstruction and classification within a unified semantic communication framework while maintaining classification performance comparable to the separate pipeline. The reconstruction gap also confirms that unified multi-task optimization introduces a practical trade-off between visual fidelity and downstream semantic utility.

### 5.3. Interpretation of the Multi-Task Loss

The composite loss used in this study provides a direct mechanism for coordinating reconstruction and classification objectives. Nevertheless, the PSNR transformation, the 30 dB reference value, the scaling factors, and the fixed weighting coefficient are engineering choices rather than theoretically optimal settings. They are used to place the reconstruction-related term and the classification loss within a numerically manageable range and to prevent either task from completely dominating the joint optimization.

In the revised experiments, the weighting coefficient is fixed at a = 0.5 to represent a balanced setting. The observed results indicate that further optimization of reconstruction quality does not necessarily lead to higher classification accuracy. Therefore, the current loss formulation should be regarded as a practical baseline for joint reconstruction–classification training rather than a final solution to multi-task loss balancing.

More advanced strategies, such as uncertainty-based weighting, gradient normalization, dynamic task weighting, or task-specific optimization schedules, may provide a more adaptive balance between the two objectives. A systematic comparison with these methods would be valuable in future work.

### 5.4. Shared Representation and Model Design

Mission-ADWITT is motivated by the assumption that image reconstruction and classification can benefit from partially shared representations. The improved classification and reconstruction results obtained after ADWITT initialization provide indirect evidence that the reconstruction backbone contains features that can support both visual recovery and semantic recognition. However, the present study does not directly visualize or quantitatively analyze the shared feature space.

Accordingly, the current results should not be interpreted as definitive proof that all shared features are equally beneficial to both tasks. Future studies may employ feature visualization, t-SNE or UMAP projection, centered kernel alignment, representation similarity analysis, or gradient interaction analysis to examine how reconstruction-oriented and classification-oriented information is distributed within the shared backbone.

ResNet18 is selected as the classification branch because it is a widely used and relatively lightweight architecture with stable optimization behavior. Using a standard classifier also helps isolate the effects of semantic communication and the multi-task training strategy. Nevertheless, the present experiments do not establish that ResNet18 is the optimal classification backbone. Comparisons with lightweight alternatives, such as MobileNet, or stronger classification architectures could further clarify whether the observed behavior is architecture-dependent.

### 5.5. Limitations

Several limitations should be acknowledged. First, the revised evaluation focuses on CIFAR-10 under AWGN channels. This controlled setting allows a complete comparison among the separate baseline, the without-initialization variant, and the baseline-initialized Mission-ADWITT model, but it does not fully represent larger image resolutions, more diverse datasets, or more complicated fading channels.

Second, the current study reports deterministic full-test-set results for the available checkpoints, but repeated independent training runs, standard deviations, and confidence intervals are not included. Therefore, the reported improvements describe the observed model instances and should not be interpreted as a complete statistical significance analysis.

Third, although inference delay is reported, a full deployment-oriented complexity evaluation, including FLOPs, parameter count, memory consumption, and throughput, is not provided. The delay values of the compared methods remain within the same order of magnitude, and the current results are insufficient to claim comprehensive computational superiority.

Fourth, the ablation study primarily evaluates the effect of ADWITT backbone initialization. It does not independently isolate all possible factors, such as the PSNR transformation, shared feature extraction, alternative classification backbones, or adaptive task-weighting strategies. The current ablation therefore provides focused evidence for the importance of initialization, but not a complete decomposition of every architectural and optimization component.

Finally, Mission-ADWITT remains an incremental extension of the ADWITT framework. Its contribution is primarily the formulation and evaluation of a unified reconstruction–classification semantic communication system rather than the introduction of a fundamentally new physical-layer transmission architecture.

### 5.6. Future Work

Future work will investigate dynamic loss-balancing strategies to reduce the reconstruction–classification trade-off and improve robustness under different task priorities. More flexible feature-sharing mechanisms, task-specific adapters, and partially decoupled reconstruction and classification branches may also help preserve reconstruction quality while maintaining semantic classification performance.

Further experiments will consider larger and more diverse image datasets, additional wireless channel models, and repeated training runs to evaluate cross-dataset generalization and statistical stability. Feature-space analysis will also be conducted to provide more direct evidence of task interaction and shared semantic representations. In addition, computational complexity and deployment metrics will be examined more systematically to evaluate the practical applicability of the proposed framework.

## 6. Conclusions

This study presented Mission-ADWITT, a multi-task semantic communication framework for joint image reconstruction and classification. The framework extends the ADWITT wireless image transmission backbone with a ResNet18-based classification branch and jointly optimizes reconstruction fidelity and classification performance through a composite multi-task objective. The purpose of the proposed framework is not to replace reconstruction-specialized semantic communication models, but to investigate the feasibility of supporting image recovery and downstream semantic recognition within a unified receiver-side system.

The revised experiments demonstrate that reconstruction-pretrained initialization is important for the joint optimization of Mission-ADWITT. Compared with the Mission-ADWITT variant without ADWITT initialization, the baseline-initialized model improves the average classification accuracy from 75.156% to 82.988%, the average PSNR from 27.933 dB to 30.828 dB, and the average MS-SSIM from 0.9637 to 0.9804. The average multi-task loss is also reduced from 102.970 to 58.883. These results indicate that initializing the reconstruction backbone from a pretrained ADWITT model provides a more stable starting point for joint reconstruction–classification fine-tuning.

Compared with the separate ADWITT + ResNet18 baseline, the baseline-initialized Mission-ADWITT achieves comparable classification accuracy, with average values of 82.988% and 82.940%, respectively. However, the proposed unified model does not match the reconstruction quality of the separately optimized ADWITT baseline. Its average PSNR and MS-SSIM remain lower than those of the separate reconstruction model. Therefore, the experimental results should not be interpreted as comprehensive superiority over the separate baseline. Instead, they demonstrate that a unified semantic communication model can maintain competitive classification performance while jointly supporting image reconstruction, although a reconstruction–classification trade-off remains.

The current study is limited to CIFAR-10 under AWGN channels and uses a fixed multi-task loss coefficient. Repeated independent training runs, confidence intervals, broader complexity analysis, and a complete decomposition of all architectural and optimization components are not included. Future work will investigate adaptive loss balancing, improved feature-sharing mechanisms, alternative classification backbones, larger and more diverse datasets, additional channel models, and more systematic statistical and computational evaluations. These extensions may help reduce the reconstruction gap while preserving the semantic advantages of joint multi-task communication.

## Figures and Tables

**Table 1:** Experimental settings for the revised CIFAR-10 evaluation.

Item	Setting
Dataset	CIFAR-10
Test samples	10,000 images per SNR
Channel model	AWGN
Training SNR	Randomly sampled from 0 to 20 dB
Evaluation SNR values	0, 5, 10, 15, and 20 dB
Channel bandwidth parameter	C = 192
Multi-task loss coefficient	a = 0.5
Optimizer	Adam
Learning rate	0.0001
Batch size	128
Fine-tuning epochs	3
Optimizer updates	3906 per epoch; 11,718 in total
Independent training runs	1
Checkpoint selection	Highest accuracy during epoch-wise evaluation; ties resolved by higher PSNR and then lower loss
Main evaluation metrics	Accuracy, PSNR, MS-SSIM, loss, delay

**Note:** The reported results are based on one formal training run; therefore, standard deviations and confidence intervals are not reported. The custom training loader repeats the 50,000-image training set ten times per epoch, resulting in 500,000 sample accesses and 3906 optimizer updates per epoch.

**Table 2:** Compared methods in the revised experiments.

Method	Description	Role
Separate ADWITT + ResNet18	ADWITT reconstructs the image, and a ResNet18 classifier performs classification separately.	Separate baseline
Mission-ADWITT without ADWITT initialization	Joint reconstruction–classification model without loading the pretrained ADWITT reconstruction backbone.	Ablation baseline
Mission-ADWITT with ADWITT initialization	Mission-ADWITT initialized with the pretrained ADWITT reconstruction backbone and jointly fine-tuned.	Proposed revised model

**Table 3:** Average CIFAR-10 performance under AWGN channels over SNR = 0, 5, 10, 15, and 20 dB.

Method	Accuracy (%) ↑	PSNR (dB) ↑	MS-SSIM ↑	Multi-Task Loss ↓	Delay (s/Sample) ↓
Separate ADWITT + ResNet18 baseline	82.94	42.209	0.9969	N/A	0.000584
Mission-ADWITT without ADWITT initialization	75.156	27.933	0.9637	102.97	0.000423
Mission-ADWITT with ADWITT initialization	82.988	30.828	0.9804	58.883	0.000447

**Note:** ↑ indicates that a higher value is better, whereas ↓ indicates that a lower value is better. N/A indicates not applicable; the separate ADWITT + ResNet18 baseline does not use the joint multi-task loss.

**Table 4:** Per-SNR CIFAR-10 performance under AWGN channels.

Method	SNR	Accuracy (%) ↑	PSNR (dB) ↑	MS-SSIM ↑	Multi-Task Loss ↓	Delay (s/Sample) ↓
Separate ADWITT + ResNet18 baseline	0	82.33	34.133	0.9899	N/A	0.000549
Separate ADWITT + ResNet18 baseline	5	82.84	38.961	0.9966	N/A	0.000555
Separate ADWITT + ResNet18 baseline	10	83.05	43.144	0.9988	N/A	0.000613
Separate ADWITT + ResNet18 baseline	15	83.22	46.426	0.9995	N/A	0.000573
Separate ADWITT + ResNet18 baseline	20	83.26	48.38	0.9997	N/A	0.00063
Mission-ADWITT without ADWITT initialization	0	74.45	26.021	0.9368	106.447	0.000422
Mission-ADWITT without ADWITT initialization	5	75.27	27.724	0.9619	102.292	0.000421
Mission-ADWITT without ADWITT initialization	10	75.4	28.42	0.9708	102.31	0.000427
Mission-ADWITT without ADWITT initialization	15	75.23	28.707	0.9741	102.088	0.000424
Mission-ADWITT without ADWITT initialization	20	75.43	28.793	0.975	101.712	0.00042
Mission-ADWITT with ADWITT initialization	0	82.44	28.932	0.9678	60.81	0.000443
Mission-ADWITT with ADWITT initialization	5	83.05	30.654	0.9802	59.148	0.000455
Mission-ADWITT with ADWITT initialization	10	83.09	31.345	0.9839	58.357	0.000444
Mission-ADWITT with ADWITT initialization	15	83.15	31.565	0.9849	58.094	0.000442
Mission-ADWITT with ADWITT initialization	20	83.21	31.643	0.9853	58.007	0.000452

**Note:** ↑ indicates that a higher value is better, whereas ↓ indicates that a lower value is better. N/A indicates not applicable; the separate ADWITT + ResNet18 baseline does not use the joint multi-task loss.

**Table 5:** Ablation study of ADWITT reconstruction backbone initialization.

SNR	Accuracy Delta (With-Init—Without-Init)	PSNR Delta	MS-SSIM Delta	Loss Delta
Mean	7.832	2.895	0.0167	−44.087
0	7.99	2.912	0.031	−45.637
5	7.78	2.929	0.0182	−43.145
10	7.69	2.926	0.0131	−43.953
15	7.92	2.859	0.0108	−43.994
20	7.78	2.85	0.0103	−43.705

**Table 6:** Qualitative comparison with representative image semantic communication studies.

Study	System Focus	Receiver Output or Task	Evaluation Emphasis	Key Distinction from Mission-ADWITT
Bourtsoulatze et al. [4]	End-to-end deep joint source-channel coding for wireless image transmission	Reconstructed image	Image reconstruction quality under wireless channel conditions	Reconstruction-oriented system without an explicit receiver-side classification branch
Zhang et al. [8]	Multi-level semantic-assisted image transmission using high-level and low-level semantic information	Reconstructed image	Reconstruction quality and transmission efficiency under bandwidth constraints	Uses caption and segmentation semantics to assist reconstruction rather than jointly optimizing reconstruction and classification
Zhang et al. [9]	Shared image-feature extraction and compression for multiple intelligent tasks	Task-specific semantic outputs	Performance of multiple downstream intelligent tasks	Supports multiple task-oriented outputs but does not use the ADWITT backbone or the reconstruction–classification objective adopted in this study
Li et al. [22]	Adaptive transformer-based wireless image transmission	Reconstructed image	PSNR, MS-SSIM, and robustness under varying channel conditions	Provides the reconstruction backbone of Mission-ADWITT but does not contain a classification branch
Mission-ADWITT, this study	ADWITT reconstruction backbone combined with a ResNet18 classification branch and joint fine-tuning	Reconstructed image and predicted class label	Accuracy, PSNR, MS-SSIM, multi-task loss, and inference delay	Jointly supports reconstruction and classification with reconstruction-pretrained initialization

## Data Availability

The original contributions presented in this study are included in the article. Further inquiries can be directed to the corresponding author.
